# The Emergence of AGHE's Basic-Competencies Guidelines in Gerontology

**DOI:** 10.1093/geroni/igaa057.1812

**Published:** 2020-12-16

**Authors:** Robert Maiden

**Affiliations:** Alfred University, Alfred, New York, United States

## Abstract

This presentation will explore the development of AGHE’s major contemporary contribution: AGHE’s basic-competency guidelines and their role in program review and program enhancement. The presenter will describe in detail the steps in creating objective student learning outcomes and will explore how meeting these competencies improve instruction, self-reflection, program analysis and faculty discussion and ultimately gerontology programs. These guidelines and policies embody a living entity that is always evolving. Future iterations will be anticipated and discussed. Part of a symposium sponsored by the Geriatric Education Interest Group.

